# Cancer health literacy profile in Argentine oncology patients

**DOI:** 10.3332/ecancer.2025.1934

**Published:** 2025-06-27

**Authors:** Karen M Manzur, Ana Kohan Cortada

**Affiliations:** 1 Faculty of Health Sciences, Universidad Adventista del Plata, Libertador San Martín, CP 3103, Entre Ríos, Argentina; 2 Interdisciplinary Center for Research in Mathematical and Experimental Psychology, National Scientific and Technical Research Council (CONICET), CIIPME-CONICET, CP 1040, Buenos Aires, Argentina

**Keywords:** health literacy, patient-centred care, cancer, nutritional sciences, Argentina

## Abstract

**Introduction:**

Cancer Health Literacy is the individual’s ability to seek, understand, evaluate and use basic information and services necessary to make appropriate decisions regarding cancer prevention, diagnosis and treatment. The aim of this study was to analyse the Health Literacy Profile of Argentine cancer patients.

**Methodology:**

A non-experimental, descriptive, cross-sectional design was used. A non-probabilistic sampling method was applied and 500 adult cancer patients who provided their consent participated. A sociodemographic questionnaire and the Cancer Health Literacy Test were administered. Data were collected through mixed methods and analysed using R Studio.

**Results:**

The average Cancer Health Literacy score was 22.01 points (SD = 5.68, Mdn = 24), with 73% of patients classified at an intermediate level. Multiple linear regression analysis revealed that educational level and information-seeking behaviour regarding diet and cancer were significant contributing factors to this construct, explaining 30.6% of its variability (adjusted *R*^2^ = 0.306) with a large effect size (f^2^ = 0.44). Cancer Health Literacy was lower in patients with an incomplete secondary education or lower and higher in those who actively sought information.

**Conclusion:**

Cancer Health Literacy was associated with educational level and nutritional information-seeking behaviour. Measuring these factors in clinical practice contributes to evidence-based care.

## Introduction

Health literacy is a term that first appeared in 1974 in the United States in a report on education and health [1], it is linked to literacy and involves the knowledge, motivations and skills to access, understand, evaluate and apply health information to make judgments and decisions in everyday life related to health care, disease prevention and health promotion to maintain or improve quality of life across the course of life [2].

Health literacy is currently considered a multidimensional, complex and heterogeneous concept. First, it is multidimensional because its various definitions highlight different aspects of this construct, which broadens its understanding. Moreover, since there is an interaction between the demands of the system and the individual’s abilities to respond to them, it does not only involve the knowledge that people have, but also how this information is transformed into concrete actions that maintain and promote health.

Second, it is complex because it is content and context specific. Research was developed in different health settings, populations (patients, caregivers and parents), information acquisition channels and languages. Third, it is heterogeneous because it gives importance to the individual and society (infrastructure, policies and health system), being necessary for a systemic approach involving individuals, professionals, organisations and governmental decision-makers [3].

Regarding the level of health literacy of the population, in a study of 8,000 adults in 8 countries (Austria, Bulgaria, Bulgaria, Germany, Greece, Ireland, the Netherlands, Poland, Spain and Greece), 47% had limited health literacy and the variables associated with low levels were male sex, had financial deprivation, low social status, low educational level and advanced age [4]. In Latin America, only a few regional studies were conducted and with considerable variation in measurement methods, definitions of health literacy and levels were found, as adequate health literacy ranged from 5% to 73.3%. In addition, older adults, low socioeconomic status, low educational level and people who spoke a different language were more likely to have limited health literacy [5].

In Argentina, in 2009, health literacy was measured in 229 patients attending a university hospital, the prevalence of an inadequate health literacy level was 30.1% and was significantly associated with the educational level of the respondents [6]. Then, in 2015, this construct was measured in 80 post-partum women, 31.25% presented limited health literacy [7]. Later in 2017, 156 patients with diabetes were investigated; 39.7% presented inadequate health literacy and it was significantly associated with higher levels of glycosylated haemoglobin [8].

In the oncology setting, Cancer Health Literacy ‘is the individual’s ability to seek, understand, evaluate, and use basic information and services needed to make appropriate decisions regarding the prevention, diagnosis, and treatment of cancer’ [9, p.70]. In a systematic review, its importance in cancer clinical outcomes was grouped into nine categories: preventive behaviours, cancer knowledge, attitudes and beliefs, risk perception, information seeking, decision-making, quality of life, health status, post-treatment behaviours and communication between the patient and his or her healthcare provider [10].

The results show that inadequate health literacy is associated with lower adoption of preventive and screening behaviours, longer delays in identifying symptoms and seeking medical help, lower awareness of cancer, its prevention and treatment, deficiencies in risk perception, greater unmet information needs, fewer information-seeking behaviours, lower perceived quality of life, lower post-treatment compliance and lower perceived quality of communication and engagement between patient and healthcare provider [10]. A second systematic review concluded that worse outcomes are expected in patients with health literacy difficulties [11].

In relation to information-seeking behaviour, this responds to patients’ need for up-to-date, accurate, easy-to-understand information [12], on a continuous basis and with consistent messages among professionals [13] and high health literacy is one of the predictors of this behaviour [14–19].

In addition, an inverse association was found between health literacy and information overload, which occurs when the supply of information exceeds the subject’s processing capacity [20]. Similarly, limited health literacy was associated with increased effort, frustration, concern about information quality and difficulty in understanding information [16, 18, 21, 22].

Studies show that the percentage of oncology patients with low health literacy is variable, 30% of a sample of 347 patients [23], 32.5% in 339 patients [24] and 44% in 218 patients [25]. Given the importance of this construct in oncologic clinical outcomes and in the management of information for decision-making, added to the limited national background and the recent adaptation of the Health Literacy Test in Cancer in Argentina [26], the opportunity arises to study this construct in Argentine oncologic patients. For this reason, the aim of this study was to analyse the Health Literacy Profile of Argentine oncology patients in the year 2023.

## Methods

A quantitative approach with a non-experimental, descriptive, cross-sectional design was used. The sample consisted of Argentine patients over 18 years of age with a confirmed diagnosis of cancer, who could read and write and who gave their consent to participate in the study. Patients with a diagnosis of cancer at an advanced stage (end-of-life care), with severe psychiatric conditions (major depression, psychosis) and/or with cognitive and/or visual impairments that prevented them from answering the questionnaire were excluded. A non-probabilistic convenience sampling was used due to the difficulties in accessing this population, since it is distributed in different health institutions. In addition, not all the institutions that were invited agreed to participate in the study.

### Instrument

A sociodemographic questionnaire was used, asking gender, age, educational level and whether the patient sought nutritional information after receiving the diagnosis of cancer or during treatment. In addition, the Cancer Health Literacy Test (CHLT-30) [27] was applied, because it was designed specifically for this population. This instrument consists of 30 items, and each correct answer adds one point. The range of scores is from 0 to 30. In the Argentine adaptation, this instrument was subjected to the evaluation of 6 expert judges, a pilot test with 41 patients and then administered to a sample of 500 patients. Adequate psychometric properties were evidenced, with a content validity of Aiken’s V = 0.85 and internal consistency of KR = 0.733 [26]. In the adaptation to the Latino population, a scale was proposed that categorises as low if they obtain a score of 10 points or less, intermediate between 11 and 26 points and high if they reach between 27 and 30 points [9].

### Data collection procedure

Data collection was carried out in a comprehensive manner using both traditional paper and digital methods. Face-to-face collection was carried out in institutions related to oncology services, such as radiotherapy institutes, day hospitals for chemotherapy sessions and waiting rooms of clinical offices specialised in oncology. Likewise, contact was made with associations present in digital media, which simplified the connection with patients and the issuance of digital invitations through a Google Forum. In both modalities, an appropriate font size was assigned to facilitate the legibility of the questions. A total of 110 patients responded in paper format and 390 responded in digital format. Data collection was carried out during the year 2023.

### Data analysis procedure

First, a descriptive analysis was performed using frequencies and percentages for the variables sex, educational level, information-seeking behaviour and level of Cancer Health Literacy. For quantitative variables such as age and total Cancer Health Literacy score, measures of central tendency (Mean, Median, Mode) and Dispersion (Standard Deviation) were used. To study the influence of the variables mentioned above on the Cancer Health Literacy score, a multiple linear regression analysis was performed following the stepwise methodology. Then, a bivariate analysis was applied between the predictor variables and the dependent variable (Cancer Health Literacy score. In all cases, a statistically significant association was found. Second, the variables ordered according to their contribution to the *R*^2^value were introduced into the model. For each partial model, coefficients, standard error, *R*^2^, residuals and *F* statistic (anova) were recorded.

Once the final model was obtained, the assumptions of linearity, homoscedasticity, normal distribution of the residuals, multicollinearity (estimated using the variance inflation factor VIF) and presence of outliers were evaluated. Finally, the effected size was calculated for multiple regression (f2 = R^2^/ 1- R^2^). The interpretation followed the criteria of Cohen [28], where it is considered small if f2 = 0.02, medium if f2 = 0.15 and large if f2 = 0.35.

### Ethical aspects

The study was approved by the Review Committee of the Universidad del Salvador, the Teaching and Research Department and the Medical Management Board of the four participating institutions located in the provinces of Entre Ríos and Santa Fe. The principles of the Declaration of Helsinki were respected and patients signed the informed consent to express their willingness to participate in the research. All procedures respected the ethical guidelines for research with human beings indicated by the American Psychological Association [29], the Declaration of Helsinki of the World Medical Association [30] and Law 25.326 on the Protection of Personal Data [31].

## Results

**Figure 1.**
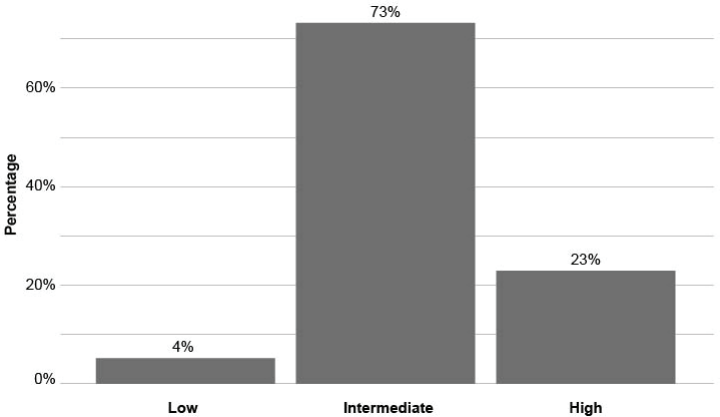
Health literacy in cancer according to scale [9].

The sample consisted of 500 oncology patients, 87.2% (*n* = 436) were women and the average age was 49.52 years (SD = 12.33). Regarding educational level, most of the participants had completed secondary education or higher (87.4%, *n* = 437), 5.6% (*n* = 28) had incomplete secondary education, 4% (*n* = 20) had completed primary education and 3% (*n* = 15) had incomplete primary education. For more details on the clinical characteristics of the patients, refer to Manzur and Kohan Cortada [32]. The patients obtained an average Cancer Health Literacy of 22.01 (SD = 5.68) points and 73% presented an intermediate level (Figure 1).

A multiple linear regression analysis was performed, following the stepwise methodology. The variables were entered into the model according to their contribution to the *R*^2^value, in the following order: educational level (*R*^2^ = 0.29), sex (*R*^2^ = 0.11), age (*R*^2^ = 0.09) and information search (*R*^2^ = 0.04) (Table 1). When the variable age was included, the variable sex and age were no longer significantly associated with the dependent variable. However, this did not occur when the nutritional information search variable was introduced. The final model with its coefficients is detailed in Table 2.

Patients with incomplete or complete primary and incomplete secondary education scored on average 6.64 points (95% CI -8.47–4.81) and 2.86 points (95% CI -4.85–0.87) lower in Cancer Health Literacy compared to those with complete secondary education. On the other hand, patients who sought information on diet and cancer after diagnosis and during treatment had on average 1.75 points (95% CI 0.60–2.90) higher Cancer Health Literacy compared to those who did not. Therefore, educational level and information-seeking behaviour explained 30.6% of the variability in Cancer Health Literacy scores (adjusted *R*^2^ = 0.306) and with a large effect size (f^2^ = 0.44).

## Discussion

In this sample of Argentine patients, 73% presented an intermediate level of health literacy in cancer, 23% high and 4% low. Other antecedents indicated higher percentages of low health literacy in these patients, for example, 30% in a sample of 347 patients [23], 32.5% in 339 patients [24] and 44% in 218 patients [25]. It is important to note that these results correspond to the general health literacy construct but in the oncologic context.

Therefore, the most appropriate comparison can be made with the study by Barros *et al* [33], which used the same instrument and cut-off points as the present investigation. In a sample of 70 oncology patients, 56.4% presented intermediate levels of health literacy in cancer, 40.8% high and 2.8% low. The results in both studies were similar, with the highest percentage of patients having an intermediate level of health literacy in cancer. However, in the Portuguese population, a higher percentage of patients with a high level was found, compared to the Argentinean sample. These differences should not be attributed initially to the educational level of the patients, since in this background 83.1% had completed secondary education or higher and, in this study, 87.4% had this condition. However, despite having similar educational levels, it is possible that differences in terms of competencies acquired during formal education may influence the results. Furthermore, these differences could be explained by the cultural adaptation of the instrument, the particularities of the country contexts (Portugal and Argentina) and the variations in the sample size of both studies (70 versus 500 patients).

On the other hand, in relation to the factors associated with low health literacy in cancer, no significant associations were found with sex and age, but significant associations were found with a low educational level, specifically with having incomplete secondary education or less. These results coincide with published precedents that used the same questionnaire [9, 27, 33]. However, in the adaptation of the instrument to the Chinese population, a partial coincidence was observed, although no differences were found with sex, as in this study, no association was found between educational level and health literacy in cancer [34]. In that antecedent, the sample consisted of 602 patients and 34.8% with a secondary education level or higher. The authors highlight that a limitation of the study is that 86.9% had the Cantonese dialect as their mother tongue, and only 13% had Mandarin. Possibly, the lack of observed differences is associated with the linguistic aspects of the study, since this relationship was identified not only for cancer health literacy, as previously mentioned, but also for health literacy in general [4–6].

In this study, regardless of their educational level, patients who sought information on diet and cancer after diagnosis presented higher health literacy in cancer compared to those who did not. These results are in line with what has been published in the scientific literature, where high health literacy is one of the predictors of this behaviour [14–19].

The European Coalition of Cancer Patients [35] points out that receiving nutritional information is a patient’s right. In Argentine legislation, this right is supported in Law 26.529, which regulates the Rights of the patient in their Relationship with Health Professionals and Institutions, and guarantees receiving comprehensive, clear and accessible information on health and treatment [36].

In the local context, 98% (*n* = 490) of patients considered it important to receive it and 91% (*n* = 455) perceived it as a necessity. However, the most consulted source was the Internet [37]. It is important that health professionals take an active role as disseminators of this information and consider cancer health literacy and the educational level of patients to perform this role adequately.

Despite the contributions made by this study, it is essential to recognise the limitations inherent to the design and execution of the research. Among the limitations identified are first, the selection of patients with non-probabilistic sampling, which could affect the representativeness of the sample and make it difficult to extrapolate the results to the target population since participation in the study was based on the voluntary response of the patients and these could have different characteristics from those who chose not to participate. Second, the use of a mixed collection methodology, which included both virtual and paper-based modalities, could have introduced potential limitations in the uniformity of responses.

## Conclusion

This research deepened our knowledge of the profile of the Argentine oncology patient by incorporating constructs such as cancer health literacy, educational level and nutritional information-seeking behaviour, aspects that had not been addressed in this way in the regional and local context.

In clinical practice, it is essential that professionals from different disciplines, such as medicine, nursing, nutrition, psychology, among others, who care for these patients, consider their educational level, particularly in those with incomplete high school education or lower level of education, since their health literacy in cancer may be limited.

Finally, incorporating into clinical practice spaces that allow the collection of information on cancer health literacy, educational level and nutritional information-seeking behaviour will make it possible to identify patients with limited cancer health literacy, adapt professional communication strategies and contribute to evidence-based oncology care.

To improve the health literacy of these patients, it is recommended that professionals provide clear and understandable information about cancer and its treatment using educational tools adapted to different levels of understanding and modalities (written brochures, videos and orientation sessions).

## List of abbreviations

SD: Standard Deviation
Mdn: Medium
Mo: Fashion

## Conflicts of interest

The authors declare that they have no conflicts of interest.

## Funding

This study did not receive funding.

## Author contributions

Both authors met the four ICMJE criteria for authorship. They contributed equally to the conception and design of the study, data analysis, drafting of the manuscript and approval of the final version. Data collection was performed by KMM.

## Figures and Tables

**Table 1.** Evolution of the multiple linear regression model on cancer health literacy in Argentine patients.

Mod	B0	B1 (PI/PC)	B2 (SI)	B3 (UI)	B4 (UC)	B2 (sex)	B3 (ed)	B4 (info)	RStd Error	*p*	*F* global	*R*^2^aj
1	20.26	−6.86	−3.15	2.69	3.96				4.76	<0.05	*F* = 53.07 <0.0001	0.294
2	18.97	−6.09	−2.73	2.55	3.79	1.51			4.745	0.04	*F* = 53.07 <0.0001	0.298
3	20.37	−5.84	−2.61	2.45	3.71	1.27	−0.02		4.744	0.256	*F* = 53.07 <0.0001	0.299
4	19.06	−5.77	−2.41	2.37	3.63	1.10	−0.02	1.62	4.71	0.006	*F* = 32.83 <0.0001	0.308

Mod: model; B0 intercept; PI/PC: incomplete/complete primary; SI: incomplete secondary; UI: incomplete university; UC: complete university; Sex: sex; Ed: age; info: information-seeking behaviour; Rstd error: standard error; *R*^2^aj: adjusted *R*^2^

**Table 2.** Final multiple linear regression model of health literacy in cancer in Argentine patients.

	Coef.	95% CI	Error standard	*t*	*p*
Intercept	18.84	17.53–20.16	0.67	28.19	<0.0001
Edu^1^. PI and PC	−6.64	−8.47–4.81	0.93	−7.14	<0.0001
Edu. SI	−2.86	−4.85–0.87	1.01	−2.82	0.005
Edu. UI	2.56	1.26–3.87	0.66	3.86	0.00013
Edu. UC	3.85	2.74–4.96	0.57	6.80	<0.0001
Information Search^2^	1.75	0.60–2.90	0.58	2.99	0.0029

1 Reference: Completed Secondary Education

2 Reference: Did not seek information
